# Evidence-Based Practice in Medical Education: Mapping a Research and Critical Analysis Program Against the Sicily Statement

**DOI:** 10.1007/s40670-022-01584-z

**Published:** 2022-07-07

**Authors:** Pippa Burns, Kathryn M Weston

**Affiliations:** Graduate School of Medicine, University of Wollongong, Wollongong, NSW Australia

**Keywords:** Evidence-based practice, Evidence-based medicine, Education, Medical, Program development, Curriculum

## Abstract

This paper describes how evidence-based practice (EBP) is taught through an integrated curriculum across a 4-year graduate MD program. Mapping of the curriculum to the domains of the *Sicily Statement* of EBP was an effective approach to evaluate integration of EBP into a graduate medical education program. The longitudinal integration of EBP engages students in multiple opportunities to learn, understand, and apply these concepts. The EBP program incorporates both traditional and innovative teaching approaches and can easily be adapted for other professional courses. This whole-course approach is graduating a new generation of doctors with a sound understanding of EBP.

## Background

One of the challenges in medical education is the availability of contemporary frameworks against which to map the curriculum. The teaching of skills and application of evidence-based practice (EBP) is one area where this challenge needs to be addressed.

Inclusion of EBP within medical curriculums has been shown to impact clinical practice [1]. Indeed, the use of research to inform clinical decision-making is now seen as a core skill for physicians [2]. This begs the question: how can medical educators ensure that an EBP curriculum that engages students is itself based on sound evidence?

This paper maps the longitudinal teaching and integration of EBP in a 4-year graduate-entry medical course against the *Sicily Statement* of EBP, a consensus statement from the second international conference of Evidence-Based Health Care Teachers and Developers [3]. The authors of the Sicily Statement make the case that a curriculum grounded by five principles of EBP (Table 1) will produce health care professionals with the ability to “gain, assess, apply and integrate new knowledge […] throughout their professional lives” [3].

**Table 1:**
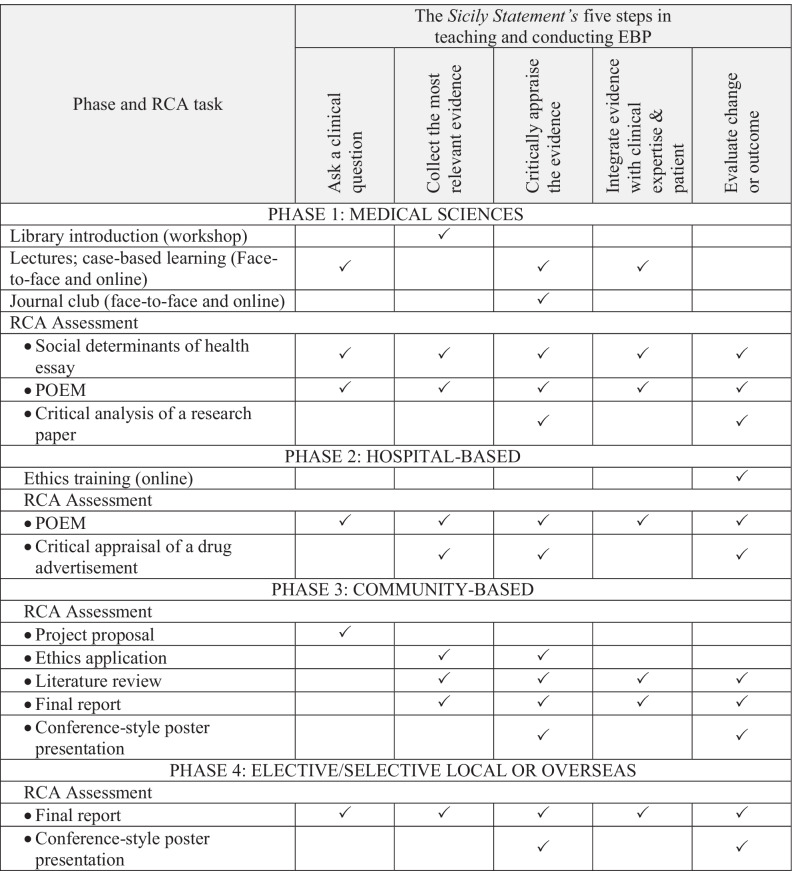
Mapping of the RCA Program against the *Sicily Statement* five steps of evidence-based practice

*POEM* Patient-Oriented Evidence that Matters

✓ indicates that this component of the RCA curriculum incorporates feature(s) that satisfy the Sicily Statement principle(s)

## Activity

The MD program discussed here comprises four curriculum themes: clinical skills, personal and professional development, medical sciences, and research and critical analysis (RCA) [4]. The RCA theme aims to graduate medical students with the capacity to critically appraise evidence and undertake independent research [4]. RCA course content integrates with other curriculum themes, teaching students fundamental research skills within the medical and clinical contexts. Thus, students learn to find, appraise, interpret, and apply evidence to their clinical practice. Skill development occurs through a mixture of methods including face-to-face lectures, a student-led journal club, individual community-based research projects, and multiple assessment tasks.

The course has four phases: Phase 1 (18 months) uses case-based learning linked to body systems and is based in the university setting [4]. Phase 2 is 12 months of conventional hospital rotations across medicine, obstetrics and gynaecology, paediatrics, psychiatry, and surgery. Phase 3, also 12 months, is spent on a longitudinal integrated clerkship placement in a community in New South Wales, Australia. The final 6 months of the medical degree (Phase 4) allows students to engage in elective, selective, and pre-internship rotations.

## Results and Discussion

Components of the RCA curriculum across the 4 years of the course were mapped against the five core activities listed in the *Sicily Statement* (Table 1).

### Statement 1: Ask a Clinical Question

Asking a clinically relevant question is represented across all phases of the RCA curriculum (Table 1). In Phase 1, students are required to submit a paper describing the impact of a social determinant on Aboriginal health. Students are required to locate literature, including grey literature, and explore the inter-play between the social determinant and health status.

Additionally, the POEM (Patient-Oriented Evidence that Matters) assessment tasks in Phases 1 and 2 specifically engage students in finding the answers to a clinical question that may be posed by a patient during a consultation [5]. Students evaluate meta-analyses to answer questions, such as *What are the advantages of selective serotonin reuptake inhibitors in treating anxiety and depression?*; and *Are new oral anti-coagulants safe to use in patients with impaired renal function?.*

During Phases 3 and 4, students undertake two research activities of their own choosing. In Phase 3, students in collaboration with a research mentor, develop a research question relevant to their placement community. They write a research proposal, complete a human research ethics application, undertake data collection and analysis, and disseminate their findings through oral presentations to their peers, academic staff, community-based health professionals, and in some cases members of the community. Evaluation has consistently shown that this authentic learning experience improves research capability among medical students [6]. A recent addition has been the Phase 4 capstone project relating to each student’s clinical experience. This task provides students with the opportunity to work on clinical focused research further expanding their experience from the community-focused research they undertook in Phase 3. A number of formats are acceptable: case report, visual clinical medicine, short research report, professional practice, or narrative piece—each of which has been designed to mirror relevant journal submission guidelines. This facilitates the pathway for students to publish their work. This capstone project has been enthusiastically embraced by the students with some still engaging with RCA staff after graduation to work on their journal submissions.

### Statement 2: Collect the Most Relevant Evidence

As well as opportunities through the POEM and research projects in Phase 3 and 4, students in Phase 2 are presented with a drug advertisement and required to investigate both implicit and explicit claims through a written assessment task. This task aims to increase students’ awareness of regulations around pharmaceutical advertising and its potential influence on prescribing habits of doctors [7]. During Phases 3 and 4, collecting evidence is a fundamental task required for completion of the student’s research reports.

### Statement 3: Critically Appraise the Evidence

Throughout the medical course, students are taught to critically appraise the evidence (Table 1). Aspects of EBP such as study design and statistics are taught through the case-based learning structure. For instance, during the female reproduction fortnight, the RCA topic of clinical trials is presented and illustrated with both historical examples (thalidomide) and current topics (human papillomavirus vaccination). Students participate in a journal club, with their peers and academic staff, where they critically appraise a scientific article, via group presentation. A summative assessment also requires critical analysis of a current research paper.

During Phase 3, students complete a literature review on their research topic, drawing on previously taught skills including critical appraisal. Critical appraisal of their research also forms a part of the final report. High achieving students are encouraged to publish and present their work. In the first 10 years of the program, more than 80 publications and presentations have resulted from 656 student projects, representing a 12.2% publication rate. This result is similar to an analysis of publications from a public health research experience at a New Zealand university where 11.1% of projects resulted in publication or presentation [8]. It is important to note, however, that research publications and presentations are only one way of evaluating a research curriculum. Defining core research competencies is a challenge for medical educators [9] and evaluating how graduates locate and use evidence in later professional practice is difficult.

### Statement 4: Integrate the Evidence with Clinical Expertise, Patient Preferences, and Values to Make a Practice Decision

As students’ clinical expertise and skills in EBP grow during the course, they are able to use their research and critical appraisal skills within their clerkship locations. Community-based preceptors have recognised the skills of Phase 3 students in researching topics of relevance, and contributing to improvements in processes, including provision of care [10]. The case-based learning and research activities are scaffolded to allow students to progressively develop their skills, ensuring that the nexus between scientific evidence and their own clinical expertise, patient preferences, and values promotes provision of quality care. Over 85% of senior medical students select either the case study or visual clinical report format for their capstone project [11], reflecting both this statement and the students’ emerging interest in clinical research.

### Statement 5: Evaluate Change or Outcome

Evaluation is critical to implementing change. Published reports from this program demonstrate the following: increased research competency in students [6]; positive impacts on students in a clinical setting [10]; opportunities for students to research areas of national health priority [12]; and that upon graduation students are work-ready, research-competent doctors [13]. Moreover, the design of the RCA component of this medical course can easily be adapted to other professional courses [13].

Previous research has demonstrated a need for clinical academics to better understand research ethics [14]. During Phase 2, students undertake online training on human research ethics. This ensures that students meet university guidelines for ethical research enabling them to undertake their own research during Phases 3 and 4.

At an individual level, summative assessments are important for students to recognise their level of understanding. Students also have the opportunity to engage with academic staff through formative assessments prior to the summative assessment submission.

The 4-year RCA curriculum discussed in this paper has been mapped to all five steps of the *Sicily Statement* (Table 1). This contrasts with a recent report that most medical courses only cover two to four of the five steps [15]. The mapping activity has shown that the *Sicily Statement*, developed from expert opinion, is an effective tool for evaluation of EBP within a medical curriculum.

One interesting feature of the RCA curriculum is that it includes the development of students’ understanding of the ethical conduct of research and provides an opportunity for them to participate in the research ethics process. To our knowledge, the ethical conduct of research has not previously been explicitly included in teaching of EBP. Beyond the practical aspects of students being compliant with university legislation, the authors argue that an understanding of good ethical research practice is paramount in respecting participants, and ensuring that principles of beneficence, justice, research merit, and integrity are maintained in all research activities [16].

The RCA program has been developed to build students’ knowledge and understanding of research and EBP. It uses a mix of traditional and innovative approaches through which to teach EBP, a feature that is in line with recent reviews investigating the best methods of teaching EBP [17, 18]. Importantly, the design of the RCA component of this medical course can easily be adapted to other professional courses [13]. Through this whole-of-course approach, a new generation of doctors are graduating with a sound understanding of the importance of EBP and the capacity and confidence to engage with and create new evidence. This, in turn, may lead to better patient care.

## References

[1] Straus SE, Ball C, Balcombe N, Sheldon J, McAlister FA (2005). Teaching evidence-based medicine skills can change practice in a community hospital. J Gen Intern Med.

[2] Patelarou A (2017). Approaches to teach evidence-based practice among health professionals: an overview of the existing evidence. Adv Med Educ Pract.

[3] Dawes M (2005). Sicily statement on evidence-based practice. BMC Med Educ.

[4] Mullan (2017). ‘Involve me and I learn’: development of an assessment program for research and critical analysis. J Med Educ Curric Dev.

[5] Slawson DC, Shaughnessy AF (2000). Becoming an information master: using POEMs to change practice with confidence. J Fam Pract.

[6] Mullan JR, Weston KM, Rich WC, McLennan PL (2014). Investigating the impact of a research-based integrated curriculum on self-perceived research experiences of medical students in community placements: a pre- and post-test analysis of three student cohorts. BMC Med Educ.

[7] Goldacre B. Bad pharma: how medicine is broken, and how we can fix it. Harper Collins Publishers; 2013.

[8] Al-Busaidi IS, Tarr GP (2018). Dissemination of results from medical student public health research training and factors associated with publication. Postgrad Med J.

[9] Lee MG, Hu WC, Bilszta JL (2020). Determining expected research skills of medical students on graduation: a systematic review, to Medical Science Educator. Med Sci Educ.

[10] Weston KM, Hudson JN (2014). Clinical scholarship among preceptors supervising longitudinal integrated medical clerkships in regional and rural communities of practice. Aust J Rural Health.

[11] Burns P, Weston K, Rich W, Akhund S, Mullan J, McLennan P. Developing students’ skills and experience in writing for publication. 2019 [Online]. Available: https://anzahpe.org/resources/Documents/Conference/Past%20Conference%20documentation/2019%20Proceedings.pdf.

[12] Weston K, Mullan J, Rich W, McLennan P (2018). Medical student research during a longitudinal community-based placement can provide opportunities for learning about public health. Educ Sci.

[13] Weston KM, Mullan JR, Rich WC, Crowther S, Bushnell JA, McLennan PL (2017). Graduating work-ready professionals: research competency as a critical curriculum component. Curric Teach.

[14] Weston KM (2016). Academic guidance in medical student research: how well do supervisors and students understand the ethics of human research?. J Acad Ethics.

[15] Larsen CM, Terkelsen AS, Carlsen AMF, Kristensen HK (2019). Methods for teaching evidence-based practice: a scoping review. BMC Med Educ.

[16] The National Health and Medical Research Council, the Australian Research Council and Universities Australia. National Statement on Ethical Conduct in Human Research 2007 (Updated 2018). Commonwealth of Australia, Canberra. https://www.nhmrc.gov.au/about-us/publications/national-statement-ethical-conduct-human-research-2007-updated-2018#block-views-block-file-attachments-content-block-1

[17] Kyriakoulis K (2016). Educational strategies for teaching evidence-based practice to undergraduate health students: systematic review. J Educ Eval Heal Prof.

[18] Young T, Rohwer A, Volmink J, Clarke M (2014). What are the effects of teaching evidence-based health care (EBHC)? Overview of systematic reviews. PLoS ONE.

